# Fabrication of AgCl/Ag_3_PO_4_/graphitic carbon nitride heterojunctions for enhanced visible light photocatalytic decomposition of methylene blue, methylparaben and *E. coli*

**DOI:** 10.1039/d0ra09147b

**Published:** 2021-02-04

**Authors:** Haishuai Li, Linlin Cai, Xin Wang, Huixian Shi

**Affiliations:** Institute of New Carbon Materials, College of Material Science and Engineering, Taiyuan University of Technology Taiyuan 030024 China shihuixian@tyut.edu.cn

## Abstract

Herein, a novel ternary nanocomposite AgCl/Ag_3_PO_4_/g-C_3_N_4_ was successfully synthesized *via* sedimentation precipitation and ion exchange method. The photocatalytic performance of the as-prepared AgCl/Ag_3_PO_4_/g-C_3_N_4_ nanocomposite was investigated *via* photocatalytic degradation of methylene blue (MB), methylparaben (MPB) and inactivation of *E. coli* under visible light irradiation. The AgCl/Ag_3_PO_4_/g-C_3_N_4_ composite presented the optimal photocatalytic performance, degrading almost 100% MB and 100% MPB, respectively. The excellent stability of AgCl/Ag_3_PO_4_/g-C_3_N_4_ was also verified in the cycle operations; the degradation efficiency of MPB could still be maintained at 85.3% after five cycles of experiments. Moreover, the AgCl/Ag_3_PO_4_/g-C_3_N_4_ composite displayed more superior photocatalytic inactivation efficiency with 100% removal of *E. coli* (7-log) in 20 min under visible light irradiation. The efficient photo-generated charge separation originated from a strong interaction in the intimate contact interface, which was confirmed by the results of photocurrent and EIS measurements. In addition, radical trapping experiments revealed that hole (h^+^) was the predominant active species in the photocatalytic system. Based on the experimental results, a photocatalytic mechanism for the degradation of parabens over AgCl/Ag_3_PO_4_/g-C_3_N_4_ was also proposed. We believe that this work provides new insights into the multifunctional composite materials for the applications in solar photocatalytic degradation of harmful organic compounds and common pathogenic bacteria in wastewater.

## Abstract 2

A noval ternary nanocomposite AgCl/Ag_3_PO_4_/g-C_3_N_4_ was successfully synthesized for photocatalytic degradation of methylene blue, methylparaben and inactivation of *E. coli* under visible light irradiation, showing excellent photocatalytic degradation performance and stability.

## Graphical abstract

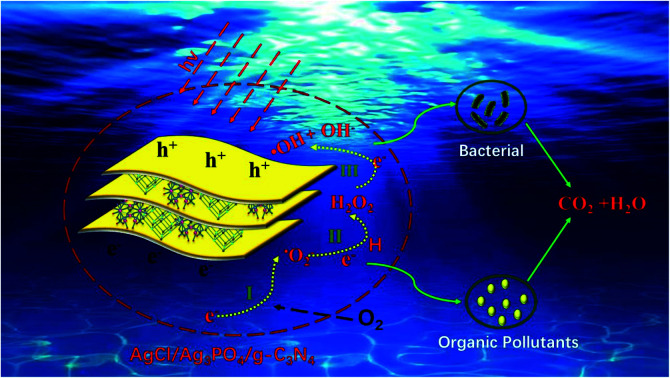

## 1. Introduction

Water pollution has become a serious hazard to public health and ecosystems in the world.^1–3^ The composition of wastewater contains many kinds of toxic chemicals, including organic dyes, endocrine disrupting compounds (EDCs), and common pathogenic bacteria, and various strategies have been explored for solving the issue.^1,4^ The increasing emergence of organic dyes from textile and food industries has become one of the most important types of water contaminants. Among the EDCs, methylparaben (MPB) with estrogenic or androgenic activity at very low concentrations may cause potential and real detrimental effects on the endocrine systems of humans and wildlife.^5,6^ Moreover, infectious diseases caused by the harmful waterborne pathogens threaten the public health. According to the latest report of the World Health Organization, about 884 million people in the world use untreated water, which make waterborne diseases the leading cause of death.^7,8^ Therefore, it is a great challenge to eliminate toxic chemicals and disease-causing waterborne pathogens using a single material, simultaneously. One way of achieving this objective is to explore new bifunctional nanocomposites capable of efficiently degrading organic pollutants and eradicating pathogenic bacteria *via* an eco-friendly technique.^9,10^

Over the past decades, photocatalytic technology has been widely regarded as the most promising technology to solve environmental pollution and energy shortage issues.^11,12^ Semiconducting photocatalysts, especially those with high catalytic efficiency and good stability under visible light irradiation, have been widely applied in the degradation of organic contaminants owing to their ability to directly harvest solar energy and excellent high-visible-light-driven photocatalytic activities.^13^

Recently, Ag-based photocatalysts have been widely reported for the treatment of persistent pollutants in wastewater, such as AgX (X = Cl, Br, and I), Ag_3_CO_3_, Ag_3_PO_4_ and CdS, which show a much faster photodegradation rate than the conventional TiO_2_.^14–17^ Among them, silver orthophosphate (Ag_3_PO_4_), an efficient n-type photocatalytic material, has high quantum efficiency (up to 90%) and indirect band gap of 2.36 eV, making it a prospective visible-light induced photocatalyst.^18,19^ However, there are still some drawbacks that largely limit the practical application of Ag_3_PO_4_, including recombination of electron–hole pairs and severe photo-corrosion, which restricts the reusability of the Ag_3_PO_4_ composite photocatalysts.^20–22^ Besides, Ag_3_PO_4_ is also prone to photogeneration to form metallic Ag. The black metallic silver particles suspended in the reaction system, inevitably shield the absorption of visible light, thereby reducing the photoactivity during the photocatalytic reaction.^20,22^ To address these issues, diverse techniques have been proposed to synthesize novel and more efficient visible-light-driven photocatalysis materials. It has been proved that metallic Ag and plasmon-induced Ag@AgX (X = Cl, Br, I) nanoparticles on the surface of Ag_3_PO_4_ can effectively enhance the photoactivity and stability of Ag_3_PO_4_.^23^ Hence, AgCl was introduced to improve its photocatalytic performance.^24^ Among the reported photocatalytic materials, polymeric carbon nitride (CN) is an efficient and stable metal-free organic polymer material.^25^ Graphitic carbon nitride (g-C_3_N_4_), a well-known π-conjugated material, has received incessant interest in photocatalysis due to its visible light absorption, low cost, environmental long-term stability.^26–28^ Generally speaking, cheap organic precursors containing carbon and nitrogen elements can be prepared by thermal condensation to prepare g-C_3_N_4_.^29,30^ Its unique chemical composition and conjugated electronic structure endows it with strong nucleophilic capability. The typical layered structure of g-C_3_N_4_ can provide a supportive surface for Ag_3_PO_4_ to disperse and synthesize semiconductor heterojunctions. In addition, the conduction band (CB) and valence band (VB) edge of g-C_3_N_4_ are more negative than the negative band of Ag_3_PO_4_, which inhibits the secondary recombination of Ag_3_PO_4_ and promotes electron transfer. Previous reports on Ag-based semiconductor photocatalysts including g-C_3_N_4_/Ag_3_PO_4_–H_2_O_2_,^31^ g-C_3_N_4_/Ag_3_PO_4_/NCDs,^32^ g-C_3_N_4_/Ag_2_CO_3_,^33^ and Ag_2_O/g-C_3_N_4_ (ref. 34) have shown them to exhibit superior photocatalytic performance.^35^

Herein, we present the preparation and characterization of AgCl/Ag_3_PO_4_/g-C_3_N_4_ ternary composites as a photocatalyst for the degradation of methylene blue (MB), methylparaben (MPB) and the inactivation of bacteria. The ternary composites enable more visible light harvesting and a large contact area for fast interfacial photo-generated charge separation and photocatalytic reactions. The photocatalytic performance and stability of the composites were investigated and bactericidal performance of the samples was evaluated by time-killing study.

## 2. Experimental

### 2.1 Synthesis of samples

All materials (analytical purity) were obtained from Macklin and used without further purification.

#### Synthesis of g-C_3_N_4_

80 mmol of melamine was placed in a vacuum tube furnace, heated to 550 °C in a muffle furnace and maintained for 4 h, and then cooled to room temperature naturally. The product was washed several times with deionized water and ethanol to remove the soluble reactants and impurities. After drying in vacuum at 60 °C, a yellow agglomerate was obtained and ground into powder for further use.

#### Synthesis of Ag_3_PO_4_

5 mmol NaH_2_PO_4_ was dissolved in 50 mL deionized water at room temperature, and then 0.015 mmol AgNO_3_ was added dropwise to the NaH_2_PO_4_ solution. The color of the solution changed to yellow and after stirring for 5 h, the obtained yellow Ag_3_PO_4_ precipitate was washed, and then dried at 60 °C to obtain Ag_3_PO_4_ nanoparticles.

#### Synthesis of AgCl/Ag_3_PO_4_

1 g Ag_3_PO_4_ was dispersed in 50 mL deionized water by ultrasonication, then 0.1 M NaCl solution was dropped into the Ag_3_PO_4_ dispersion, and after vigorously stirring for 2 h the precipitate was collected, washed and dried. The final photocatalyst AgCl/Ag_3_PO_4_ was obtained.

#### Synthesis of AgCl/Ag_3_PO_4_/g-C_3_N_4_

**Scheme 1:**
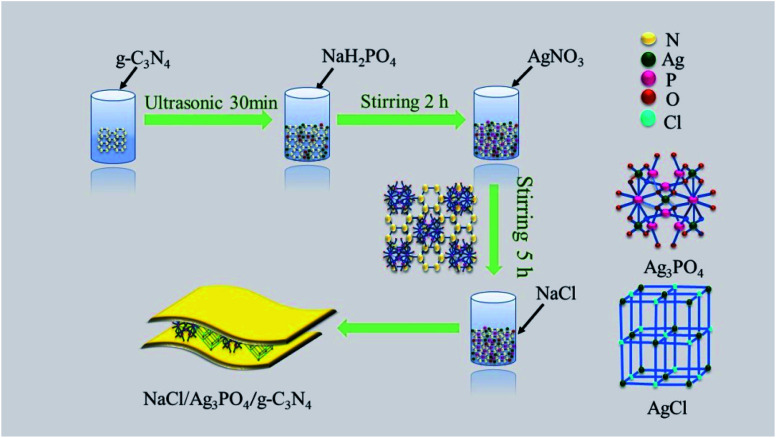
Synthetic procedure for AgCl/Ag_3_PO_4_/g-C_3_N_4_.

0.2 M g-C_3_N_4_ solution was added to the Ag_3_PO_4_/AgCl solution under mechanical stirring, followed by continuous stirring for 5 h. Subsequently, the precipitate was collected, washed with water and ethanol to neutrality in a low-speed centrifuge and dried in an electro thermal blast drying oven at 75 °C. Finally, the product was collected, ground and labeled as AgCl/Ag_3_PO_4_/g-C_3_N_4_. The schematic diagram of the preparation process is shown in Scheme 1.

### 2.2 Characterization of the photocatalyst

The crystallographic properties of AgCl/Ag_3_PO_4_/g-C_3_N_4_ composites were characterized on a DX-2700 X-ray diffractometer (XRD) at a scanning step size of 0.03° in the 2*θ* range of 20 to 80°. The surface morphology of the AgCl/Ag_3_PO_4_/g-C_3_N_4_ composite was characterized by scanning electron microscopy (JSM-7800F) and transmission electron microscopy (JEOL JEM-2010). Ultraviolet-vis (UV-vis) diffuse reflectance spectra were measured on a UV-vis Cary 50 Bio with a scanning step in the wavelength range of 200–800 nm. The photoluminescence (PL) spectra were investigated on a F-4600 spectrophotometer with an excitation wavelength of 390 nm. The XPS analyses were carried out on a K-Alpha Photoelectron Spectrometer with an X-ray source of Al Kα (*hv* = 1253.6 eV). All the binding energies were calibrated internally by C 1s at 284.8 eV. The photocurrent and resistance measurements were performed on an electrochemical workstation (CHI660E, CHI Instruments, Inc., China).

### 2.3 Photocatalytic degradation experiments

The photocatalytic activities of resultant nanocomposites were estimated by the degradation of MB, MPB and *E. coli* under visible light irradiation.

In a typical procedure, 50 mg photocatalyst was dispersed in 100 mL 20 mg L^−1^ MB and MPB solution. The light source was a 300 W metal-halide lamp (PLS-SXE300, Shanghai Bilang Co., Ltd., China) with a UV cut-off filter (*λ* < 400 nm) and the illumination intensity was kept *ca.* 10 mW cm^−2^. In all experiments, the temperature of the reaction was maintained at 25 ± 1 °C by the water continuously circulated in the jacket surrounding the reactor. Before irradiation, all the reaction samples were stirred for 30 min in dark to obtain an adsorption–desorption equilibrium. Afterwards, aliquots (2 mL) of dispersion was collected and filtered at regular intervals of the irradiation time, and the MB and MPB concentrations were detected by using a UV-vis spectrophotometer at the wavelengths of 235 and 369 nm, respectively. For the assessment of photocatalytic activities of the resultant photocatalyst, the degradation efficiency was calculated by *C*/*C*_0_ × 100%, where *C* and *C*_0_ are the concentrations of MB and MPB at a real-time *t* and the initial concentration, respectively. For comparison, light (without photocatalyst) and dark controls (without light) were also performed.

The photocatalytic disinfection was carried out using a 300 W xenon lamp with a cut-off filter of 420 nm and the illumination intensity at around 10 mW cm^−2^. All glass apparatuses were autoclaved at 120 °C for 20 min for the disinfection experiments to ensure sterility. After incubation in 10% nutrient broth solution at 30 °C for 18 h with shaking, the bacterial cell was washed with sterilized saline. The cell density was adjusted to 1.5 × 10^7^ colony forming units per milliliter (cfu mL^−1^). After the photocatalytic treatment, an aliquot of the reaction solution (5 mL) was taken out at different time and immediately diluted with sterilized saline solution (0.9% NaCl). The appropriate dilution of the sample was spread on the nutrient agar and incubated for 24 h at 37 °C. All the experiments were performed in triplicates.

#### Fluorescence spectroscopy

Each aliquot of bacteria collected before and after the photocatalytic treatment at different time was stained with typical cell-labeling dye mixtures of SYTO 9 (a green-fluorescent nucleic acid dye) according to the recommended procedure in the bacterial viability kit to detect living and dead bacterial cells, respectively. After the incubation in the dark at 25 °C for 15 min, the samples were transferred to cover slips. Fluorescence spectroscopy of the samples was performed with a fluorescence microscope (Nikon ECCLIPSE 80i, Japan), which was equipped with a Spot-K slide CCD camera (Diagnostic Instruments Inc., USA) and a filter block N UV-2A consisting of excitation filter Ex 400–680 (Nikon, Japan). A FIT with the intensity of 100 mW m^−2^ was selected as the visible light source.

## 3. Results and discussion

### 3.1 Morphology and structure characterization of materials

#### SEM and EDS mapping

**Fig. 1:**
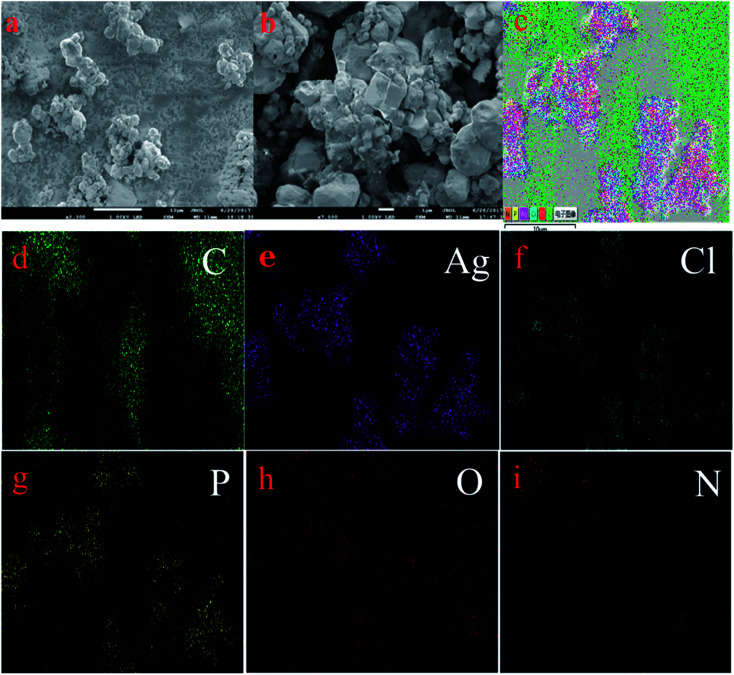
SEM images of Ag_3_PO_4_/AgCl/g-C_3_N_4_ (a and b), EDS mapping (c) and corresponding elemental mapping images of Ag_3_PO_4_/AgCl/g-C_3_N_4_ (d–i).

Morphological features of the samples were examined by SEM and EDS mapping images and the corresponding results are shown in Fig. 1. It could be found clearly (Fig. 1a) that the structure of AgCl/Ag_3_PO_4_ particles was regular ellipse, and g-C_3_N_4_ act as a support for both AgCl and Ag_3_PO_4_ particles, when it was introduced into the AgCl/Ag_3_PO_4_ composites; the composite displayed an agglomerated bulk with a rough surface, which could enhance the surface area and led to a better photocatalytic performance.^36^ The elemental mapping images (Fig. 1c–i) displayed the uniform distribution of elements C, Ag, Cl, P, O and N in the Ag_3_PO_4_/AgCl/g-C_3_N_4_ composite.

#### XRD

**Fig. 2:**
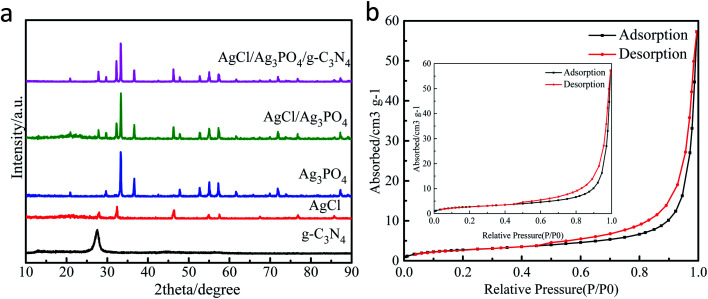
XRD pattern of samples (a); N_2_ adsorption–desorption isothermal curve and pore size distribution of AgCl/Ag_3_PO_4_/g-C_3_N_4_ (b).

The crystalline structure and phase composition information of the as-prepared samples were further characterized by XRD. As shown in Fig. 2, strong diffraction patterns of the Ag_3_PO_4_ sample was indexed to the cubic structure of Ag_3_PO_4_ and the 2 theta values at 20.8°, 29.7°, 33.3°, 36.6°, 47.8°, 52.7°, 55.0°, 57.3° and 71.2° were indexed to the (110), (200), (210), (211), (310), (222), (320), (321), and (421) crystal planes, respectively, of Ag_3_PO_4_ (JCPDS no. 06-0505).^37^ For pure AgCl, the diffraction peaks located at 2 theta = 27.8°, 32.2°, 46.2°, 54.8°, 57.5°, 67.5°, 76.7°, and 85.7° corresponding to (111), (200), (220), (311), (222), (400), (420) and (422) planes can be assigned to the cubic phase of crystalline AgCl (JCPDS no. 31-1238.).^38^ The diffraction peak at 27.6° can be attributed to the (110) plane of g-C_3_N_4_.^18^ It was clear that the AgCl/Ag_3_PO_4_/g-C_3_N_4_ composite consisted of AgCl, Ag_3_PO_4_ and g-C_3_N_4_ phases, indicating successful synthesis of a high-purity composite.

#### BET

N_2_ adsorption–desorption test was performed to study the textural properties of the materials. Fig. 2 shows the nitrogen adsorption/desorption isotherms of AgCl/Ag_3_PO_4_/g-C_3_N_4_, and Table 1 shows the BET specific surface areas obtained based on the results shown in Fig. 2. The isotherms of AgCl/Ag_3_PO_4_/g-C_3_N_4_ are of type-II classification and it has the typical hysteresis loops of mesoporous materials. At the same time, it can be concluded from the pore size distribution diagram that the pore size distribution is 80–110 nm, which shows that we have prepared a ternary mesoporous material to greatly improve its structural stability.

**Table 1:** List of specific surface area, pore volume and pore diameter of materials

Name	BET/(m^2^ g^−1^)	Pore volume/(cm^3^ g^−1^)	Average aperture/(Å)
AgCl/Ag_3_PO_4_/g-C_3_N_4_	10.1424	0.0885	34.9157

#### XPS

**Fig. 3:**
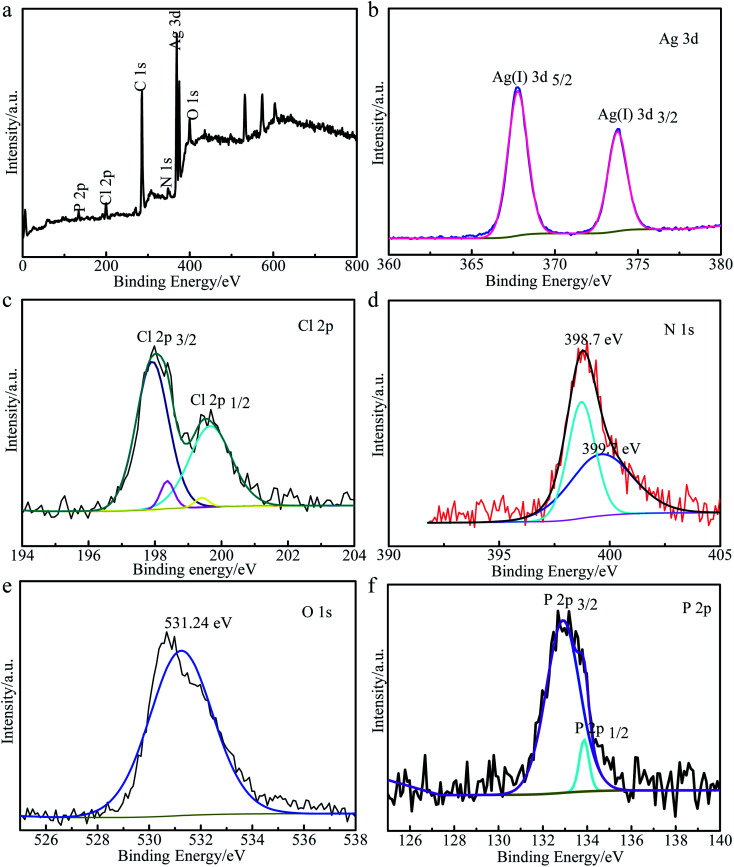
XPS spectra of AgCl/Ag_3_PO_4_/g-C_3_N_4_: (a) survey spectra; (b) Ag 3d spectra; (c) Cl 2p spectra; (d) N 1s spectra; (e) O 1s spectra and (f) P 2p spectra.

To further investigate the surface elemental composition and the valence states of Ag_3_PO_4_/AgCl/g-C_3_N_4_ composite, XPS was carried out. It can be seen from Fig. 3a that the sample is composed of P, Cl, C, N, O and Ag elements, which is consistent with the results of EDS mapping. In the Ag 3d spectrum (Fig. 3b), two peaks were observed at 367.77 and 373.77 eV in the Ag 3d region, which were assigned to Ag 3d_5/2_ and Ag 3d_3/2_ orbitals typical of Ag(i), respectively.^39^ The binding energy peaks of Cl 2p (Fig. 3c) at 198.08 and 199.68 eV were associated with Cl 2p_3/2_and Cl 2p_1/2_ of Cl^−^ in AgCl.^40,41^ In addition, N 1s (Fig. 3d) showed a peak at 398.8 eV, and two photoelectron peaks were fitted at 398.7 eV and 399.7 eV, which were derived from the sp^2^ hybridized N in C=N–C and the sp^3^ hybridized N in N–[C]_3_.^42^ Additionally, as shown in Fig. 3e, the peak at 531.24 eV could be assigned to O 1s of the lattice oxygen of Ag_3_PO_4_.^43^ As shown in Fig. 3f, two individual bands at 132.8 and 133.4 eV could be attributed to the electron orbitals of P 2p_3/2_ and P 2p_1/2_, respectively.

### 3.2 Optical and electrical properties of the material

#### UV-vis and band gap analyses

**Fig. 4:**
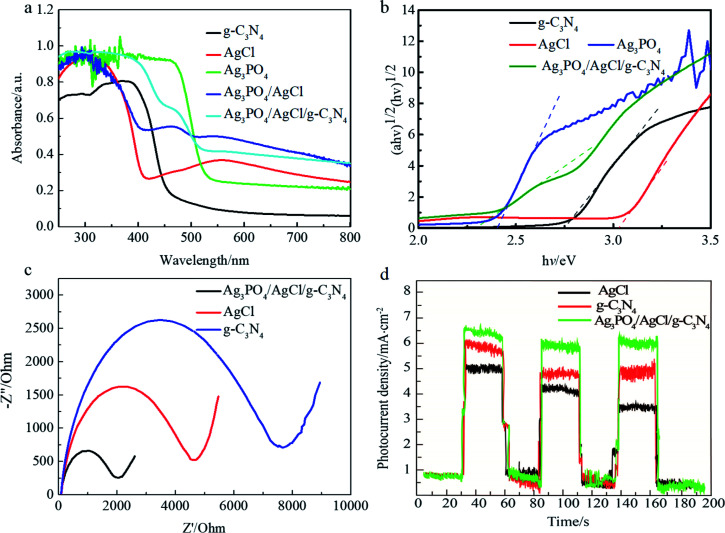
(a) UV-vis spectra of different samples, (b) plot of (*αhv*)^1/2^*versus* energy of different samples, (c) EIS plots of the samples under irradiation with visible light and (d) transient photocurrent response of samples.

The optical properties of the AgCl/Ag_3_PO_4_/g-C_3_N_4_ composite were recorded using UV-vis spectroscopy and the results are displayed in Fig. 4a. The absorption band edge of pure Ag_3_PO_4_ was found at around 530 nm, and the absorption capability was diminished at wavelengths >530 nm.^11,44,45^ The light absorption capability of g-C_3_N_4_ is weak in both UV and visible regions. When g-C_3_N_4_ was introduced into the Ag_3_PO_4_/AgCl composite, the band edge of the absorption spectra also showed an obvious red-shift, and the visible light absorption capacity in the range of 400–500 nm was further enhanced, resulting in the promotion of photocatalytic activity. The absorption of Ag_3_PO_4_/AgCl/g-C_3_N_4_ in the visible light region is apparently enhanced, which can be attributed to the interaction between the valence band and conduction band of g-C_3_N_4_ and Ag_3_PO_4_. Compared to the pure Ag_3_PO_4_, AgCl and g-C_3_N_4_, the AgCl/Ag_3_PO_4_/g-C_3_N_4_ composite photocatalyst has a stronger absorption capacity for visible light, improving the conversion efficiency of visible light and further improving the photocatalytic activity of the composite material. Moreover, the band gap energy can be estimated from a plot of (*αhv*)^1/2^*versus* photo energy (*hv*). The *x*-intercept of the tangent line gives an approximation of the band gap energy of the samples. As shown in Fig. 4b, the band gap energy of the AgCl/Ag_3_PO_4_/g-C_3_N_4_ composite is about 2.3 eV, which is smaller than that of AgCl and Ag_3_PO_4_, indicating that the AgCl/Ag_3_PO_4_/g-C_3_N_4_ composite can respond better in the visible region of 540 nm.

#### Photoelectrochemical (PEC) measurements

Photoelectrochemical properties were measured to investigate the excitation and transfer of photo-generated carriers and the interface reaction ability of the charges in photocatalytic materials. Generally, the smaller arc size reflects smaller charge transfer resistance at the interface.^46^ In Fig. 4c, the AgCl/Ag_3_PO_4_/g-C_3_N_4_ composite shows the smallest radius of arc, suggesting that the heterostructure has a lower resistance and the fastest interface charge transfer as well as the best photo-generated carrier separation efficiency. Additionally, Fig. 4d displays the current–time (*I*–*t*) curves of AgCl/Ag_3_PO_4_/g-C_3_N_4_, AgCl and g-C_3_N_4_ with several 30 s light on/off cycles. Compared to g-C_3_N_4_ and AgCl/Ag_3_PO_4_ composite, the ternary composite AgCl/Ag_3_PO_4_/g-C_3_N_4_ showed an increased photocurrent response. The formation of photocurrent is mainly by the separation and diffusion of photo-generated electron–hole pairs from the internal structure of the photocatalyst to its surface and free charge acceptors in the electrolyte.^47^ Therefore, the increased photocurrent of AgCl/Ag_3_PO_4_/g-C_3_N_4_ indicates more efficient separation and less recombination of the photo-generated electron–hole pairs, which is conducive to enhance photocatalytic performance.

### 3.3 Photocatalytic degradation and disinfection performance

**Fig. 5:**
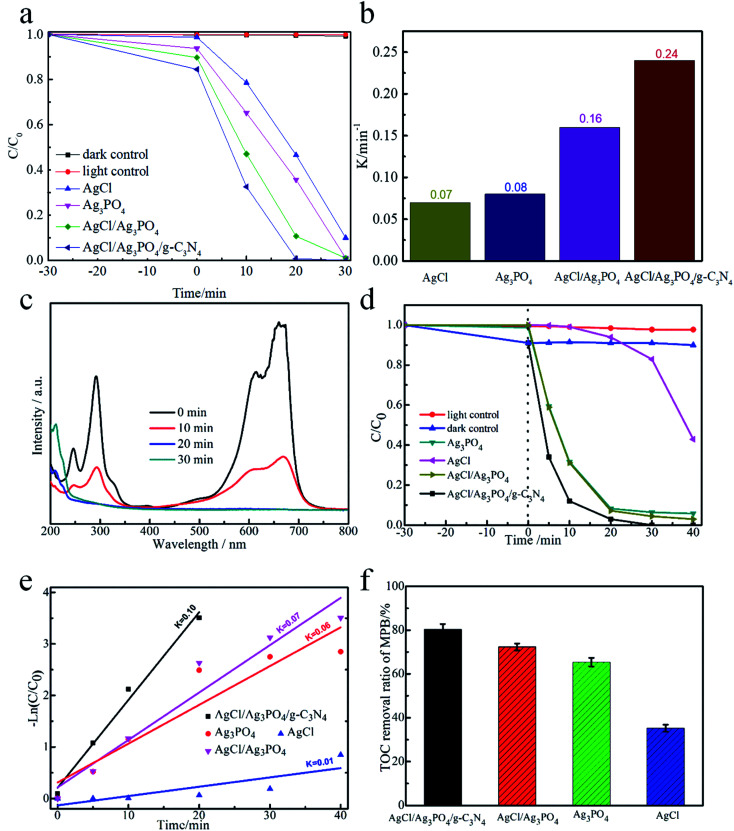
The photocatalytic degradation activity for MB over different samples under visible light irradiation (a); reaction kinetic rate constant of as-prepared samples (b); time-dependent absorption spectra of MB under visible light irradiation (c); the photocatalytic performance for MPB over different samples under visible light irradiation (d), reaction kinetic rate constant of as-prepared samples (e), TOC of MPB removal efficiency (f).

The photocatalytic performance of AgCl/Ag_3_PO_4_/g-C_3_N_4_ composite was evaluated by the photocatalytic degradation of MB and MPB under visible-light irradiation. Fig. 5a demonstrate that 91% of MB could be degraded by AgCl within 30 min, while Ag_3_PO_4_ and AgCl/Ag_3_PO_4_ could completely degrade MB within 30 min; however, after combining with g-C_3_N_4_, the AgCl/Ag_3_PO_4_/g-C_3_N_4_ composite showed enhanced photocatalytic activity, leading to 100% degradation of MB within 20 min. To quantitatively explore the corresponding MB degradation kinetic curves of the samples, the data were matched with a first-order model. As shown in Fig. 5b, the pseudo-first-order rate constants (*k*) of AgCl, Ag_3_PO_4_, AgCl/Ag_3_PO_4_ and AgCl/Ag_3_PO_4_/g-C_3_N_4_ composites were calculated to be 0.07, 0.08, 0.16 and 0.24 min^−1^, respectively. The *k* value of the AgCl/Ag_3_PO_4_/g-C_3_N_4_ composite was about 3.42 and 3.4 times higher than that of pure AgCl and Ag_3_PO_4_, respectively. Moreover, the time-dependent UV-vis absorption spectra of the photocatalytic degradation of MB by the AgCl/Ag_3_PO_4_/g-C_3_N_4_ composite was studied, as shown in Fig. 5c, with the absorption peak at about 660 nm corresponding to the characteristic absorption of MB. After 10 min of visible light irradiation, the intensity of the absorption peak at 660 nm was significantly decreased, and with further increase of the illumination duration, the absorption peak at 660 nm fades away. The results indicate that the AgCl/Ag_3_PO_4_/g-C_3_N_4_ composite can completely oxidize parabens under visible light.

The samples was also estimated by degradation of MPB to evaluate the photocatalytic capacity of the AgCl/Ag_3_PO_4_/g-C_3_N_4_ composite, the samples were also estimated by the degradation of MPB. As shown in Fig. 5d, only very little amount of MPB was degraded in the dark and in the absence of catalyst, indicating that MPB was considerably stable and self-photolysis can be neglected. Among Ag_3_PO_4_, AgCl, AgCl/Ag_3_PO_4_ and AgCl/Ag_3_PO_4_/g-C_3_N_4_ composite, the AgCl/Ag_3_PO_4_/g-C_3_N_4_ composite exhibited the highest photocatalytic performance, and about 100% of MPB was degraded within 30 min; in contrast, 94.8%, 92.3% and 60% of MPB was degraded within 40 min by AgCl/Ag_3_PO_4_, Ag_3_PO_4_ and AgCl, respectively. The excellent photocatalytic performance of AgCl/Ag_3_PO_4_/g-C_3_N_4_ could be ascribed to the high migration efficiency of the photo-induced electron–hole pairs. As shown in Fig. 5e, the rate constant for the photocatalytic degradation of MPB with AgCl/Ag_3_PO_4_/g-C_3_N_4_ was 0.1 min^−1^, which was 1.43, 1.67, and 10 times higher than those with AgCl/Ag_3_PO_4_ (0.07 min^−1^), Ag_3_PO_4_ (0.06 min^−1^) and AgCl (0.01 min^−1^), respectively. We can confirm that the AgCl/Ag_3_PO_4_/g-C_3_N_4_ composite is more effective than the other samples. Additionally, the total organic carbon (TOC) experiment was further carried out to track the degradation of MPB during the photocatalytic reaction process. Fig. 5f shows the results of TOC mineralization efficiency on the photocatalytic degradation of MPB with different samples. The mineralization yield of Ag_3_PO_4_/AgCl/g-C_3_N_4_ can reach up to 80.27% within 40 min, which is higher than that of Ag_3_PO_4_/AgCl (72.3%), Ag_3_PO_4_ (68%) and AgCl (36.8%). The superiority of AgCl/Ag_3_PO_4_/g-C_3_N_4_ is understandable since it has stronger light absorption and faster charge transfer rate. This result also indicated that MPB was indeed photocatalytically degraded into inorganic substances (such as H_2_O and CO_2_). Therefore, AgCl/Ag_3_PO_4_/g-C_3_N_4_ is an excellent photocatalytic composite material that could degrade organic parabens into inorganic substances.

**Fig. 6:**
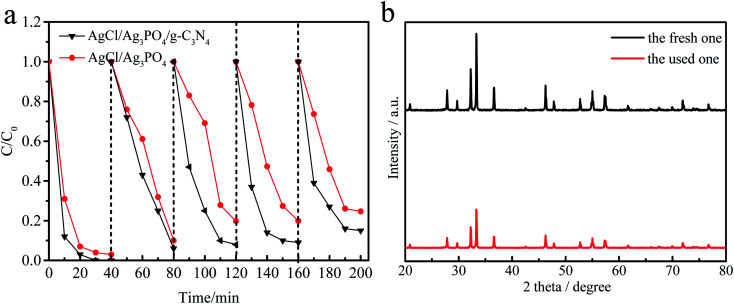
Cycling runs for MPB over AgCl/Ag_3_PO_4_ and AgCl/Ag_3_PO_4_/g-C_3_N_4_ under visible light irradiation (a), the XRD patterns of AgCl/Ag_3_PO_4_/g-C_3_N_4_ before and after use (b).

It is well-known that the stability of a photocatalyst is essential for practical applications. The recycling experiments were performed for five times for the degradation of MPB over AgCl/Ag_3_PO_4_/g-C_3_N_4_ to evaluate the photocatalytic stability. As shown in Fig. 6a, the degradation of MPB was 100%, 94.8%, 92.6%, 91.9% and 85.3%, respectively. In contrast, the degradation over AgCl/Ag_3_PO_4_ decreased from 92% to 76% after five recycling runs. It can be found that the stability of the ternary composite Ag_3_PO_4_/AgCl/g-C_3_N_4_ material with g-C_3_N_4_ as the matrix material is further improved compared to that of the Ag_3_PO_4_/AgCl. In addition, no additional characteristic peaks were observed in the XRD patterns (Fig. 6b) of Ag_3_PO_4_/AgCl/g-C_3_N_4_ after cycling, which indicates that the crystal structure did not change significantly after the photocatalytic reaction. This further proved that the sample has adequate stability without remarkable reduction of photocatalytic activity under visible light irradiation.

**Fig. 7:**
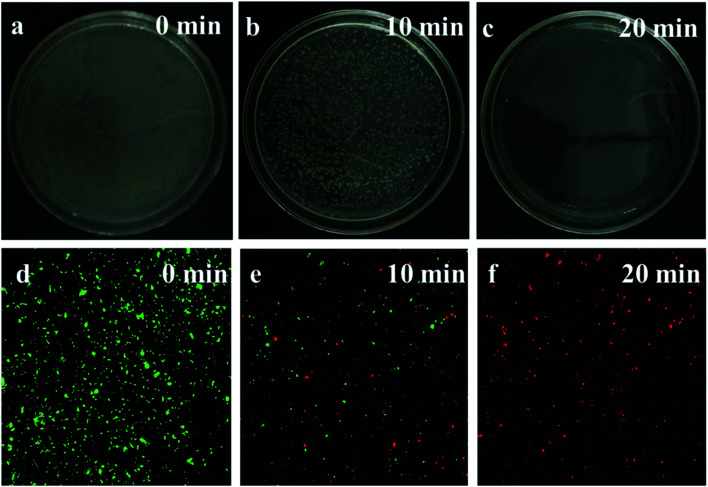
(a–c) The disinfection efficiencies of *E. coli* by AgCl/Ag_3_PO_4_/g-C_3_N_4_ at different time; (d–f) photoluminescence images of *E. coli* by AgCl/Ag_3_PO_4_/g-C_3_N_4_ at different time.

Fig. 7 shows the disinfection efficiency of *E. coli* by AgCl/Ag_3_PO_4_/g-C_3_N_4_ for different irradiation time. A mass of *E. coli* was alive without light irradiation. However, more than half of the *E. coli* was killed after being irradiated for 10 min, and all the *E. coli* was killed after 20 min irradiation. This result indicates that the AgCl/Ag_3_PO_4_/g-C_3_N_4_ composite has excellent photocatalytic anti-bacterial properties.

### 3.4 Photocatalytic mechanism analysis

**Fig. 8:**
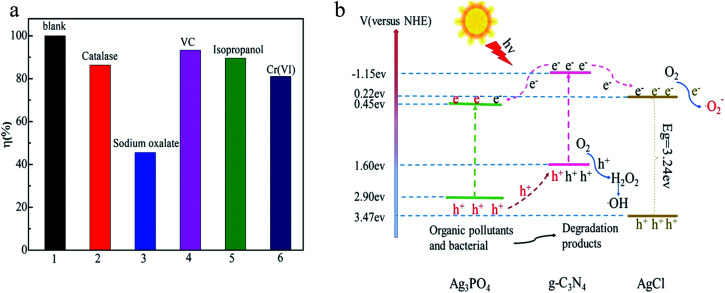
Photocatalytic inactivation efficiency of MPB by AgCl/Ag_3_PO_4_/g-C_3_N_4_ with different scavengers (a), proposed photocatalytic mechanism of the as-prepared AgCl/Ag_3_PO_4_/g-C_3_N_4_ nanocomposite (b).

In order to get insights into the photocatalytic mechanism of the Ag_3_PO_4_/AgCl/g-C_3_N_4_ composite, it is necessary to identify and assess which reactive species played the most prominent role in the photodecomposition of MPB. The trapping experiments of radicals were conducted to measure the effect of the active substances on the final degradation results. In the present work, sodium oxalate, catalase, ascorbic acid (VC), isopropanol and potassium dichromate were used as scavengers for photo-generated holes (h^+^), hydrogen peroxide (H_2_O_2_), superoxide radicals (˙O^2−^), hydroxyl radicals (˙OH) and photo-generated electrons (e^−^), respectively.^48–50^ As shown in Fig. 8a, the degradation of MPB was 34.4%, 65.6%, 88.6%, 82.1% and 83.5%, respectively, indicating that h^+^ was the main reactive species in the MPB photodecomposition process. These results demonstrate that photo-generated h^+^ are the dominant species for the decomposition of MPB, while ˙OH, ˙O^2−^, H_2_O_2_ and e^−^ played relatively minor roles in the MPB photocatalytic decomposition process.

Based on above analyses, the possible photocatalytic mechanism of the AgCl/Ag_3_PO_4_/g-C_3_N_4_ nanocomposite is shown in Fig. 8b. AgCl/Ag_3_PO_4_/g-C_3_N_4_ was excited to photogenerate electrons and holes under visible light irradiation. The staggered band gaps promote electron transfer, so g-C_3_N_4_ with a more negative CB (−1.15 eV) could easily transfer the photo-generated electrons to the CB of Ag_3_PO_4_ (+0.45 eV) and AgCl (−0.22 eV). At the same time, Ag_3_PO_4_ with more positive VB energy would transfer the photogenerated holes to the VB of g-C_3_N_4_. Because AgCl has a wide band gap and cannot be activated by visible light, it is used as an important electron acceptor to capture and shuttle electrons that would further promote the separation of electron–hole pairs of Ag_3_PO_4_ and g-C_3_N_4_.^51,52^ Therefore, the AgCl/Ag_3_PO_4_/g-C_3_N_4_ photocatalyst shows enhanced photocatalytic activity compared with pure AgCl or Ag_3_PO_4_. Under visible light irradiation, the excited photo-generated holes could directly oxidize the pollutants; it also could react with O_2_ and finally generate reactive ˙OH, which will induce the degradation of the organic pollutant. Then, the accumulated electrons on the CB could reduce the O_2_ to form superoxide radicals (˙O_2_), and then participate in photooxidation. In this way, the accumulation of electrons in the CB of Ag_3_PO_4_ can be transfer effectively, which prevents the decomposition of photo-induced corrosion and improves the stability of Ag_3_PO_4_.

1 AgCl/Ag_3_PO_4_/g-C_3_N_4_ + *hv* → h^+^ + e^−^

2 e^−^ + O_2_ → ˙O_2_^−^

3 ˙O_2_^−^ + 2H + e^−^ → H_2_O_2_

4 H_2_O_2_ + e^−^ → ˙OH + OH^−^

5 h^+^ + e^−^ + ˙O_2_^−^ + ˙OH + organic pollutant (bacteria) → CO_2_ + H_2_O

## 4. Conclusion

In summary, a novel ternary AgCl/Ag_3_PO_4_/g-C_3_N_4_ composite was prepared by loading Ag_3_PO_4_/AgCl onto g-C_3_N_4_ with a large specific surface area. The AgCl/Ag_3_PO_4_/g-C_3_N_4_ composite showed excellent photocatalytic efficiency for the removal of MB, MPB and *E. coli*. The degradation rates of MB and MPB over Ag_3_PO_4_/AgCl/g-C_3_N_4_ composite can both reach 100% within 20 min under visible light irradiation, and the degradation ratio of MPB remained 85.3% even after five cycles. Besides, it also showed good performance in the inactivation of *E. coli* within 20 min. The enhanced photocatalytic performance and stability could be ascribed to the combination of AgCl/Ag_3_PO_4_ and g-C_3_N_4_, which effectively promotes the transfer efficiency of the photogenerated carriers and inhibits the recombination of the photo-generated charge carriers during the photocatalytic reaction. Due to the larger specific surface area of g-C_3_N_4_, efficient separation of the photo-generated electron–hole can be achieved, which would improve the visible light conversion efficiency. This work not only demonstrates that the composite of Ag_3_PO_4_ and new carbon materials can enhance the photocatalytic properties and stability, but also provides an insight for the preparation of new high-performance photocatalysts.

## Authorship contribution statement

Haishuai Li conceived the experiments and wrote the manuscript. Linlin Cai wrote the manuscript. Xin Wang conducted the EIS and transient photocurrent response measurement and analysis. Huixian Shi supervised the project and reviewed the manuscript. All the author contributed to the data analyses.

## Conflicts of interest

The authors declared that they have no conflicts of interest to this work. We declare that we do not have any commercial or associative interest that represents a conflict of interest in connection with the work submitted.

## Supplementary Material
